# *N*-(3-Chloro-1,4-dioxo-1,4-di­hydro­naph­thalen-2-yl)-*N*-propionylpropionamide

**DOI:** 10.1107/S1600536813034302

**Published:** 2014-01-04

**Authors:** Nabil Idris, Ray J. Butcher, Oladapo Bakare

**Affiliations:** a Department of Chemistry, Howard University, 525 College Street NW, Washington DC 20059, USA

## Abstract

In the title mol­ecule, C_16_H_14_ClNO_4_, the four essentially planar atoms of the imide group [r.m.s. deviation = 0.0286 (11) Å] form a dihedral angle of 77.36 (13)° with the naphtho­quinone group [maximun deviation = 0.111 (2) Å for the carbonyl O atom in the naphthalene 1-position] and the two imide carbonyl groups are oriented *anti* with respect to each other. In the crystal, mol­ecules are connected by weak C—H⋯O hydrogen bonds, as well as π–π stacking inter­actions [centroid–centroid distance = 3.888 (3) Å], forming a three-dimensional network.

## Related literature   

For the synthesis and biological evaluation of imido-substituted 1,4-naphtho­quinone derivatives, see: Bakare *et al.* (2003 ▶); Berhe *et al.* (2008 ▶); Brandy *et al.* (2013 ▶). For the anti­cancer and anti­trypanosomal activity of related compounds, see: Bakare *et al.* (2003 ▶); Berhe *et al.* (2008 ▶); Khraiwesh *et al.* (2012 ▶). For a related structure, see: Butcher *et al.* (2013 ▶).
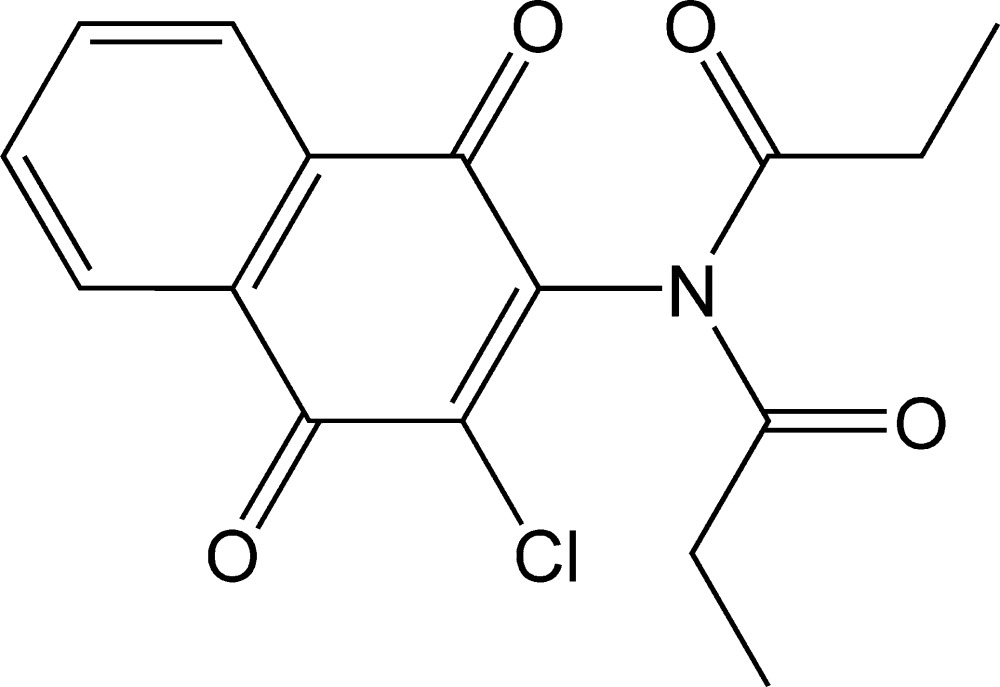


## Experimental   

### 

#### Crystal data  

C_16_H_14_ClNO_4_
*M* *_r_* = 319.73
Triclinic, 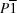

*a* = 8.1362 (9) Å
*b* = 8.2254 (9) Å
*c* = 12.4471 (11) Å
α = 98.105 (8)°
β = 92.297 (8)°
γ = 116.821 (11)°
*V* = 730.88 (15) Å^3^
*Z* = 2
Cu *K*α radiation
μ = 2.48 mm^−1^
*T* = 123 K
0.48 × 0.34 × 0.08 mm

#### Data collection  

Agilent Xcalibur (Ruby, Gemini) diffractometer
Absorption correction: multi-scan (*CrysAlis PRO*; Agilent, 2012 ▶) *T*_min_ = 0.396, *T*_max_ = 1.000
4648 measured reflections
2908 independent reflections
2419 reflections with *I* > 2σ(*I*)
*R*_int_ = 0.030

#### Refinement  

*R*[*F* ^2^ > 2σ(*F* ^2^)] = 0.042
*wR*(*F* ^2^) = 0.115
*S* = 1.01
2908 reflections
201 parameters
H-atom parameters constrained
Δρ_max_ = 0.34 e Å^−3^
Δρ_min_ = −0.30 e Å^−3^

### 

Data collection: *CrysAlis PRO* (Agilent, 2012 ▶); cell refinement: *CrysAlis PRO*; data reduction: *CrysAlis PRO*; program(s) used to solve structure: *SHELXS97* (Sheldrick, 2008 ▶); program(s) used to refine structure: *SHELXL97* (Sheldrick, 2008 ▶); molecular graphics: *SHELXTL* (Sheldrick, 2008 ▶); software used to prepare material for publication: *SHELXTL*.

## Supplementary Material

Crystal structure: contains datablock(s) I, New_Global_Publ_Block. DOI: 10.1107/S1600536813034302/lh5677sup1.cif

Structure factors: contains datablock(s) I. DOI: 10.1107/S1600536813034302/lh5677Isup2.hkl

Supporting information file. DOI: 10.1107/S1600536813034302/lh5677Isup3.cml

CCDC reference:

Additional supporting information:  crystallographic information; 3D view; checkCIF report

## Figures and Tables

**Table 1:** Hydrogen-bond geometry (Å, °)

*D*—H⋯*A*	*D*—H	H⋯*A*	*D*⋯*A*	*D*—H⋯*A*
C5—H5*A*⋯O3^i^	0.95	2.59	3.294 (2)	131
C15—H15*A*⋯O1^ii^	0.99	2.55	3.442 (2)	150
C16—H16*B*⋯O4^iii^	0.98	2.54	3.425 (3)	150
C16—H16*C*⋯O3^iv^	0.98	2.60	3.482 (3)	150

Symmetry codes: (i) 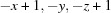; (ii) 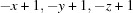; (iii) 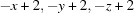; (iv) 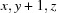.

**Fig. 1.**
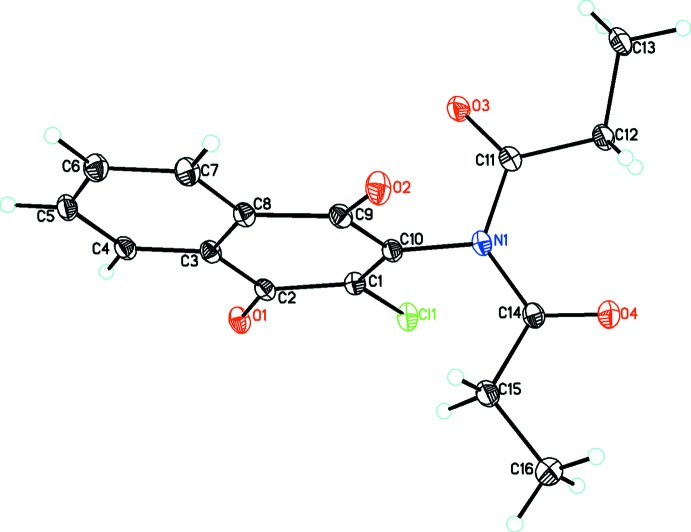
The molecular structure of the title compound with displacement parameters shown at the 30% probability level.

**Fig. 2.**
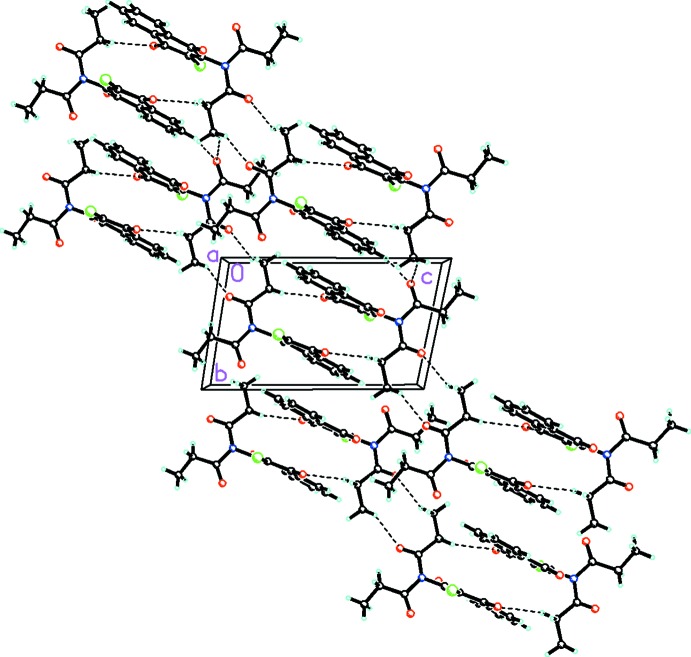
The crystal packing viewed along the a axis showing the weak C—H···O hydrogen bonds (as dashed lines) as well as the π–π stacking along the b axis.

## supplementary crystallographic information

### 1. Comment

Our group are involved in the synthesis and biological evaluation of some imido-substituted 1,4-naphthoquinone derivatives (Bakare *et al.*, 2003; Berhe *et al.*, 2008; Brandy *et al.*, 2013), and have previously reported that 2-chloro-3-dipropionylamino-1,4-naphthoquinone and some of its analogs possess inhibitory activities against certain protein kinases (Bakare *et al.*, 2003). This class of compounds have also been shown to possess anticancer (Bakare *et al.*, 2003; Berhe *et al.*, 2008) and anti-trypanosomal activities (Khraiwesh, *et al.*, 2012). As part of our studies (Butcher *et al.*, 2013) on the synthesis, properties, and structural characterization of this class of compounds, we herein present, the crystal structure of the title compound.

In the title molecule (Fig. 1), the naphthoquinone moiety deviates from planarity. The outer ring (C3-C8) is essentially planar (r.m.s. 0.004 (1) Å) while the inner ring (C1/C2/C3/C8/C9/C10) deviates slightly from planarity (r.m.s. 0.029 (1) Å) with a maximum deviation of 0.0437 (13) Å for C9. The imide group (N1/C14/O3/O4) is almost planar (r.m.s. 0.0286 (11) and the dihedral angle between this group and the whole naphthoquinone group (C1-C10/O1/O2) is 77.36 (13)°, with the two imide carbonyls oriented *anti* with respect to each other. In the crystal, molecules are linked by weak C—H···O hydrogen bonds as well as π—π interactions between the naphthoquinone rings with a centroid to centroid distance of 3.888 (3) Å between C1/C2/C3/C8/C9/C10 and C3/C4/C5/C6/C7/C8 in symmetry related rings (-*x*, 1 - *y*, 1 - *z*) forming a three-dimensional network (Fig. 2).

### 2. Experimental

The title compound was synthesized by refluxing 2-amino-3-chloro-1,4-naphthoquinone in propionyl chloride as previously reported (Bakare *et al.* (2003)). The crude compound thus obtained was crystallized from ethanol to obtain yellow crystals suitable for X-ray studies.

### 3. Refinement

H atoms were placed in geometrically idealized positions and constrained to ride on their parent atoms with a C—H distances of 0.93 and 0.97 Å *U*_iso_(H) = 1.2*U*_eq_(C) and 0.96 Å for CH_3_ [*U*_iso_(H) = 1.5*U*_eq_(C)].

### Crystal data

**Table:**

C_16_H_14_ClNO_4_	*Z* = 2
*M**_r_* = 319.73	*F*(000) = 332
Triclinic, *P*1	*D*_x_ = 1.453 Mg m^−^^3^
Hall symbol: -P 1	Cu *K*α radiation, λ = 1.54178 Å
*a* = 8.1362 (9) Å	Cell parameters from 1754 reflections
*b* = 8.2254 (9) Å	θ = 3.6–75.0°
*c* = 12.4471 (11) Å	µ = 2.48 mm^−^^1^
α = 98.105 (8)°	*T* = 123 K
β = 92.297 (8)°	Plate, colorless
γ = 116.821 (11)°	0.48 × 0.34 × 0.08 mm
*V* = 730.88 (15) Å^3^	

### Data collection

**Table:**

Agilent Xcalibur (Ruby, Gemini) diffractometer	2908 independent reflections
Radiation source: Enhance (Cu) X-ray Source	2419 reflections with *I* > 2σ(*I*)
Graphite monochromator	*R*_int_ = 0.030
Detector resolution: 10.5081 pixels mm^-1^	θ_max_ = 75.2°, θ_min_ = 3.6°
ω scans	*h* = −10→7
Absorption correction: multi-scan (*CrysAlis PRO*; Agilent, 2012)	*k* = −8→10
*T*_min_ = 0.396, *T*_max_ = 1.000	*l* = −15→15
4648 measured reflections	

### Refinement

**Table:**

Refinement on *F*^2^	Primary atom site location: structure-invariant direct methods
Least-squares matrix: full	Secondary atom site location: difference Fourier map
*R*[*F*^2^ > 2σ(*F*^2^)] = 0.042	Hydrogen site location: inferred from neighbouring sites
*wR*(*F*^2^) = 0.115	H-atom parameters constrained
*S* = 1.01	*w* = 1/[σ^2^(*F*_o_^2^) + (0.0653*P*)^2^] where *P* = (*F*_o_^2^ + 2*F*_c_^2^)/3
2908 reflections	(Δ/σ)_max_ < 0.001
201 parameters	Δρ_max_ = 0.34 e Å^−^^3^
0 restraints	Δρ_min_ = −0.30 e Å^−^^3^

### Special details

**Table:**

Geometry. All e.s.d.'s (except the e.s.d. in the dihedral angle between two l.s. planes) are estimated using the full covariance matrix. The cell e.s.d.'s are taken into account individually in the estimation of e.s.d.'s in distances, angles and torsion angles; correlations between e.s.d.'s in cell parameters are only used when they are defined by crystal symmetry. An approximate (isotropic) treatment of cell e.s.d.'s is used for estimating e.s.d.'s involving l.s. planes.
Refinement. Refinement of *F*^2^ against ALL reflections. The weighted *R*-factor *wR* and goodness of fit *S* are based on *F*^2^, conventional *R*-factors *R* are based on *F*, with *F* set to zero for negative *F*^2^. The threshold expression of *F*^2^ > σ(*F*^2^) is used only for calculating *R*-factors(gt) *etc*. and is not relevant to the choice of reflections for refinement. *R*-factors based on *F*^2^ are statistically about twice as large as those based on *F*, and *R*- factors based on ALL data will be even larger.

### Fractional atomic coordinates and isotropic or equivalent isotropic displacement parameters (Å^2^)

**Table:**

	*x*	*y*	*z*	*U*_iso_*/*U*_eq_	
Cl1	0.34840 (7)	0.42387 (7)	0.70172 (4)	0.02795 (15)	
O1	0.3577 (2)	0.2807 (2)	0.47615 (11)	0.0272 (3)	
O2	0.9546 (2)	0.4037 (2)	0.73820 (12)	0.0297 (3)	
O3	0.6017 (2)	0.1818 (2)	0.85542 (12)	0.0298 (3)	
O4	0.7362 (2)	0.7230 (2)	0.96429 (12)	0.0313 (3)	
N1	0.6911 (2)	0.4761 (2)	0.83472 (13)	0.0217 (3)	
C1	0.5193 (3)	0.3762 (3)	0.65414 (16)	0.0210 (4)	
C2	0.4900 (3)	0.2978 (3)	0.53419 (15)	0.0212 (4)	
C3	0.6266 (3)	0.2389 (3)	0.49340 (15)	0.0208 (4)	
C4	0.5978 (3)	0.1483 (3)	0.38574 (15)	0.0236 (4)	
H4A	0.4934	0.1283	0.3385	0.028*	
C5	0.7225 (3)	0.0869 (3)	0.34745 (16)	0.0254 (4)	
H5A	0.7026	0.0243	0.2742	0.031*	
C6	0.8766 (3)	0.1177 (3)	0.41683 (17)	0.0264 (4)	
H6A	0.9610	0.0751	0.3907	0.032*	
C7	0.9074 (3)	0.2096 (3)	0.52347 (16)	0.0253 (4)	
H7A	1.0137	0.2320	0.5699	0.030*	
C8	0.7822 (3)	0.2692 (3)	0.56247 (15)	0.0211 (4)	
C9	0.8146 (3)	0.3624 (3)	0.67823 (15)	0.0215 (4)	
C10	0.6669 (3)	0.4052 (3)	0.72075 (15)	0.0205 (4)	
C11	0.6645 (3)	0.3405 (3)	0.90111 (15)	0.0227 (4)	
C12	0.7214 (3)	0.4027 (3)	1.02245 (15)	0.0255 (4)	
H12A	0.6392	0.4507	1.0547	0.031*	
H12B	0.8498	0.5049	1.0359	0.031*	
C13	0.7112 (4)	0.2455 (3)	1.07811 (17)	0.0386 (6)	
H13A	0.7554	0.2926	1.1560	0.058*	
H13B	0.7892	0.1949	1.0446	0.058*	
H13C	0.5825	0.1479	1.0695	0.058*	
C14	0.7498 (3)	0.6667 (3)	0.87202 (15)	0.0223 (4)	
C15	0.8322 (3)	0.7895 (3)	0.78881 (16)	0.0268 (4)	
H15A	0.7356	0.7554	0.7270	0.032*	
H15B	0.9332	0.7671	0.7600	0.032*	
C16	0.9083 (4)	0.9933 (3)	0.83514 (19)	0.0380 (5)	
H16A	0.9591	1.0659	0.7777	0.057*	
H16B	1.0067	1.0290	0.8950	0.057*	
H16C	0.8086	1.0170	0.8627	0.057*	

### Atomic displacement parameters (Å^2^)

**Table:**

	*U*^11^	*U*^22^	*U*^33^	*U*^12^	*U*^13^	*U*^23^
Cl1	0.0231 (2)	0.0398 (3)	0.0226 (2)	0.0178 (2)	0.00071 (17)	−0.00015 (18)
O1	0.0246 (7)	0.0381 (8)	0.0195 (7)	0.0158 (7)	−0.0031 (5)	0.0034 (6)
O2	0.0244 (7)	0.0406 (9)	0.0231 (7)	0.0168 (7)	−0.0043 (6)	−0.0023 (6)
O3	0.0371 (8)	0.0241 (7)	0.0218 (7)	0.0096 (6)	−0.0017 (6)	0.0026 (6)
O4	0.0405 (9)	0.0310 (8)	0.0207 (7)	0.0158 (7)	0.0053 (6)	0.0020 (6)
N1	0.0220 (8)	0.0260 (9)	0.0156 (7)	0.0105 (7)	−0.0002 (6)	0.0018 (6)
C1	0.0184 (9)	0.0242 (9)	0.0206 (9)	0.0103 (8)	0.0026 (7)	0.0032 (7)
C2	0.0210 (9)	0.0223 (9)	0.0174 (9)	0.0072 (8)	0.0009 (7)	0.0054 (7)
C3	0.0199 (9)	0.0210 (9)	0.0182 (9)	0.0063 (7)	0.0028 (7)	0.0049 (7)
C4	0.0265 (10)	0.0235 (10)	0.0173 (9)	0.0085 (8)	0.0001 (7)	0.0045 (7)
C5	0.0317 (11)	0.0244 (10)	0.0165 (9)	0.0101 (9)	0.0040 (8)	0.0021 (7)
C6	0.0262 (10)	0.0295 (10)	0.0249 (10)	0.0134 (9)	0.0066 (8)	0.0054 (8)
C7	0.0222 (9)	0.0289 (10)	0.0229 (10)	0.0103 (8)	0.0011 (7)	0.0038 (8)
C8	0.0207 (9)	0.0223 (9)	0.0182 (9)	0.0080 (8)	0.0015 (7)	0.0037 (7)
C9	0.0198 (9)	0.0231 (9)	0.0197 (9)	0.0084 (8)	−0.0003 (7)	0.0042 (7)
C10	0.0219 (9)	0.0203 (9)	0.0168 (9)	0.0078 (8)	0.0016 (7)	0.0030 (7)
C11	0.0206 (9)	0.0267 (10)	0.0200 (9)	0.0099 (8)	0.0025 (7)	0.0053 (8)
C12	0.0280 (10)	0.0265 (10)	0.0180 (9)	0.0091 (8)	0.0009 (8)	0.0045 (7)
C13	0.0584 (15)	0.0346 (12)	0.0186 (10)	0.0182 (11)	−0.0043 (10)	0.0059 (8)
C14	0.0199 (9)	0.0270 (10)	0.0190 (9)	0.0104 (8)	−0.0003 (7)	0.0030 (7)
C15	0.0297 (10)	0.0287 (11)	0.0206 (9)	0.0122 (9)	0.0022 (8)	0.0048 (8)
C16	0.0511 (14)	0.0274 (12)	0.0303 (11)	0.0141 (11)	0.0047 (10)	0.0038 (9)

### Geometric parameters (Å, º)

**Table:**

Cl1—C1	1.7117 (19)	C7—C8	1.392 (3)
O1—C2	1.214 (2)	C7—H7A	0.9500
O2—C9	1.217 (2)	C8—C9	1.486 (3)
O3—C11	1.206 (3)	C9—C10	1.492 (3)
O4—C14	1.205 (2)	C11—C12	1.507 (3)
N1—C14	1.416 (3)	C12—C13	1.523 (3)
N1—C11	1.425 (2)	C12—H12A	0.9900
N1—C10	1.425 (2)	C12—H12B	0.9900
C1—C10	1.339 (3)	C13—H13A	0.9800
C1—C2	1.504 (3)	C13—H13B	0.9800
C2—C3	1.480 (3)	C13—H13C	0.9800
C3—C4	1.394 (3)	C14—C15	1.510 (3)
C3—C8	1.404 (3)	C15—C16	1.512 (3)
C4—C5	1.394 (3)	C15—H15A	0.9900
C4—H4A	0.9500	C15—H15B	0.9900
C5—C6	1.395 (3)	C16—H16A	0.9800
C5—H5A	0.9500	C16—H16B	0.9800
C6—C7	1.384 (3)	C16—H16C	0.9800
C6—H6A	0.9500		
			
C14—N1—C11	126.40 (16)	N1—C10—C9	116.65 (16)
C14—N1—C10	120.34 (16)	O3—C11—N1	117.25 (17)
C11—N1—C10	113.08 (16)	O3—C11—C12	123.88 (18)
C10—C1—C2	123.02 (17)	N1—C11—C12	118.84 (17)
C10—C1—Cl1	121.46 (15)	C11—C12—C13	111.82 (17)
C2—C1—Cl1	115.52 (14)	C11—C12—H12A	109.3
O1—C2—C3	122.90 (17)	C13—C12—H12A	109.3
O1—C2—C1	120.67 (17)	C11—C12—H12B	109.3
C3—C2—C1	116.41 (16)	C13—C12—H12B	109.3
C4—C3—C8	119.68 (18)	H12A—C12—H12B	107.9
C4—C3—C2	119.45 (17)	C12—C13—H13A	109.5
C8—C3—C2	120.85 (17)	C12—C13—H13B	109.5
C3—C4—C5	119.94 (18)	H13A—C13—H13B	109.5
C3—C4—H4A	120.0	C12—C13—H13C	109.5
C5—C4—H4A	120.0	H13A—C13—H13C	109.5
C4—C5—C6	119.90 (18)	H13B—C13—H13C	109.5
C4—C5—H5A	120.1	O4—C14—N1	121.10 (18)
C6—C5—H5A	120.1	O4—C14—C15	123.97 (18)
C7—C6—C5	120.52 (18)	N1—C14—C15	114.92 (16)
C7—C6—H6A	119.7	C14—C15—C16	113.04 (17)
C5—C6—H6A	119.7	C14—C15—H15A	109.0
C6—C7—C8	119.80 (18)	C16—C15—H15A	109.0
C6—C7—H7A	120.1	C14—C15—H15B	109.0
C8—C7—H7A	120.1	C16—C15—H15B	109.0
C7—C8—C3	120.15 (18)	H15A—C15—H15B	107.8
C7—C8—C9	118.99 (17)	C15—C16—H16A	109.5
C3—C8—C9	120.86 (17)	C15—C16—H16B	109.5
O2—C9—C8	122.72 (18)	H16A—C16—H16B	109.5
O2—C9—C10	119.76 (18)	C15—C16—H16C	109.5
C8—C9—C10	117.51 (16)	H16A—C16—H16C	109.5
C1—C10—N1	122.45 (17)	H16B—C16—H16C	109.5
C1—C10—C9	120.90 (17)		
			
C10—C1—C2—O1	−177.09 (19)	Cl1—C1—C10—N1	0.3 (3)
Cl1—C1—C2—O1	3.8 (3)	C2—C1—C10—C9	0.8 (3)
C10—C1—C2—C3	4.7 (3)	Cl1—C1—C10—C9	179.78 (14)
Cl1—C1—C2—C3	−174.35 (14)	C14—N1—C10—C1	−76.6 (2)
O1—C2—C3—C4	−4.5 (3)	C11—N1—C10—C1	107.9 (2)
C1—C2—C3—C4	173.66 (17)	C14—N1—C10—C9	103.9 (2)
O1—C2—C3—C8	177.06 (19)	C11—N1—C10—C9	−71.6 (2)
C1—C2—C3—C8	−4.8 (3)	O2—C9—C10—C1	174.07 (19)
C8—C3—C4—C5	0.5 (3)	C8—C9—C10—C1	−6.1 (3)
C2—C3—C4—C5	−177.96 (17)	O2—C9—C10—N1	−6.5 (3)
C3—C4—C5—C6	−0.5 (3)	C8—C9—C10—N1	173.41 (16)
C4—C5—C6—C7	−0.3 (3)	C14—N1—C11—O3	175.03 (18)
C5—C6—C7—C8	1.1 (3)	C10—N1—C11—O3	−9.8 (2)
C6—C7—C8—C3	−1.0 (3)	C14—N1—C11—C12	−6.9 (3)
C6—C7—C8—C9	178.09 (18)	C10—N1—C11—C12	168.24 (17)
C4—C3—C8—C7	0.2 (3)	O3—C11—C12—C13	6.7 (3)
C2—C3—C8—C7	178.70 (18)	N1—C11—C12—C13	−171.24 (19)
C4—C3—C8—C9	−178.88 (17)	C11—N1—C14—O4	−19.9 (3)
C2—C3—C8—C9	−0.4 (3)	C10—N1—C14—O4	165.21 (18)
C7—C8—C9—O2	6.6 (3)	C11—N1—C14—C15	158.81 (18)
C3—C8—C9—O2	−174.25 (19)	C10—N1—C14—C15	−16.0 (2)
C7—C8—C9—C10	−173.25 (17)	O4—C14—C15—C16	3.9 (3)
C3—C8—C9—C10	5.9 (3)	N1—C14—C15—C16	−174.85 (18)
C2—C1—C10—N1	−178.69 (17)		

### Hydrogen-bond geometry (Å, º)

**Table:**

*D*—H···*A*	*D*—H	H···*A*	*D*···*A*	*D*—H···*A*
C5—H5*A*···O3^i^	0.95	2.59	3.294 (2)	131
C15—H15*A*···O1^ii^	0.99	2.55	3.442 (2)	150
C16—H16*B*···O4^iii^	0.98	2.54	3.425 (3)	150
C16—H16*C*···O3^iv^	0.98	2.60	3.482 (3)	150

Symmetry codes: (i) −*x*+1, −*y*, −*z*+1; (ii) −*x*+1, −*y*+1, −*z*+1; (iii) −*x*+2, −*y*+2, −*z*+2; (iv) *x*, *y*+1, *z*.
